# ﻿*Simonia* gen. nov., a new spider genus (Araneae, Theridiosomatidae) from Southeast Asia

**DOI:** 10.3897/zookeys.1185.104120

**Published:** 2023-11-30

**Authors:** Jianshuang Zhang, Hao Yu, Yucheng Lin

**Affiliations:** 1 School of Life Sciences, Guizhou Normal University, Guiyang, Guizhou 550025, China; 2 Key Laboratory of Bio-resources and Eco-environment (Ministry of Education), College of Life Sciences, Sichuan University, Chengdu, Sichuan 610064, China; 3 The Sichuan Key Laboratory for Conservation Biology of Endangered Wildlife, Sichuan University, Chengdu, Sichuan 610064, China

**Keywords:** Indo-Burma, key, new combination, new species, ray spiders, Sundaland, taxonomy, Theridiosomatinae

## Abstract

A new theridiosomatid genus, *Simonia* Yu & Lin, **gen. nov.**, is described, with *Baalzebubyouyiensis* Zhao & Li, 2012 (♂♀, China, Vietnam and Laos) as the type species. Three species are included in *Simonia***gen. nov.**, i.e., *S.youyiensis* (Zhao & Li, 2012) **comb. nov.** ex. *Baalzebub*, *S.steineri* Yu & Lin, **sp. nov.** (♀, Huapan, Laos), and *S.sumatra* Yu & Lin, **sp. nov.** (♀, Sumatra, Indonesia). A key to theridiosomatid genera endemic to the Oriental Realm and a key to species of the new genus are provided, as well as diagnoses, descriptions and a distribution map for the species of *Simonia***gen. nov.**

## ﻿Introduction

Theridiosomatidae Simon, 1881 is a small spider family with 137 extant species in 20 genera which are mainly distributed in tropical and subtropical regions, including 28 species in 11 genera recorded in China (WSC 2023). Most theridiosomatids from the Oriental Realm have been well studied, described in detail alongside high-quality illustrations, allowing easy recognition thanks to several reviews and revisions (Miller et al. 2009; Chen 2010; Dou and Lin 2012; Zhao and Li 2012; Labarque and Griswold 2014; Lin et al. 2014; Feng and Lin 2019; Suzuki et al. 2020, 2022; Lin et al. 2022; Yang et al. 2022; Zhang et al. 2023). Despite the above-mentioned, the taxonomic relationships among some genera, such as *Baalzebub* Coddington, 1986 and its closest-related genera (e.g., *Karstia* Chen, 2010 and *Sennin* Suzuki, Hiramatsu & Tatsuta, 2022) are not yet well defined (Suzuki et al. 2022). And debate on the monophyly and delimitation of *Baalzebub* remains open, with much long-running dispute about genus placements of some *Baalzebub* species (Coddington 1986; Suzuki et al. 2022). Further, *Baalzebubsensu lato* has an unusual distribution, occurring disjunctively in the Neotropical Realm, Australia (Queensland) and southern China (WSC 2023). In addition, too many morphologically different species are placed in *Baalzebubsensu lato* indicating that the genus is definitely not monophyletic.

While examining spiders collected from Laos and Vietnam, we came across some specimens which are reported here as belonging to two new species. Both the new species possess several morphological characters shared with *Baalzebubyouyiensis* Zhao & Li, 2012, a known species widespread from Laos to Guangxi Zhuang Autonomous Region of southern China. These three species share a set of characters that distinguish them from other theridiosomatid genera, especially from *Baalzebubsensu stricto* (e.g., the generotype, *B.baubo* Coddington, 1986 and its related species from Neotropical Realm). Therefore, we are describing *Simonia* Yu & Lin, gen. nov., to accommodate the three species endemic to Southeast Asia. The goal of this paper is to provide a description of the new genus and two new species as well as redescription of *B.youyiensis* chosen as a type species of new genus.

## ﻿Materials and methods

Specimens were examined and measured with a Leica M205 C stereomicroscope. Further details were studied with an Olympus BX43 compound microscope. Copulatory organs were examined after they were dissected and detached from the bodies. Epigynes were removed and treated with lactic acid before being photographed. All specimens were preserved in 95% ethanol. Photos were taken with a Canon EOS 60D wide zoom digital camera (8.5 megapixels) mounted on an Olympus BX43 stereomicroscope. The images were montaged using Helicon Focus ver. 3.10 (Khmelik et al. 2006) image stacking software. All measurements in the paper are in millimetres. Leg measurements are given in the following sequence: total length (femur, patella, tibia, metatarsus, and tarsus).

The distribution map was generated with ArcGIS ver. 10.5 (Environmental Systems Research Institute, Inc.).

Abbreviations used in the text and figures are as follows:

**AER** anterior eye row

**Co** conductor

**CB** copulatory bursa

**CD** copulatory duct

**CL** cymbial lobe

**Cy** cymbium

**EA** embolic apophysis

**ED** embolic division

**EL** embolic lobe

**ET** embolic terminal

**FD** fertilization duct

**MA** median apophysis

**MOQ** median ocular quadrangle

**MOQA**MOQ anterior width

**MOQL** length of MOQ

**MOQP**MOQ posterior width

**Pc** paracymbium

**PER** posterior eye row

**SB** spermathecal base

**Sc** scape

**SCy** cymbial setae

**SD** sperm duct

**SH** spermathecal head

**SP** spermatheca

**SS** spermathecal stalk

**St** subtegulum

**Te** tegulum

**TF** transverse fold of epigynal plate

All material examined is deposited in the Institute of Zoology, Chinese Academy of Sciences (**IZCAS**) in Beijing and the Natural History Museum of Sichuan University (**NHMSU**) in Chengdu, China.

## ﻿Taxonomy

**Family Theridiosomatidae Simon, 1881**

### ﻿ Simonia

Taxon classificationAnimaliaAraneaeTheridiosomatidae

Yu & Lin gen. nov.

6EC9AD55-5005-5CCE-86EB-CC3CB81E9F20

https://zoobank.org/8C4A3F96-C21A-493B-B2B0-B13C284BA3EE

#### Type species.

*Baalzebubyouyiensis* Zhao & Li, 2012 (from Guangxi, China).

#### Etymology.

The generic epithet is named after the French arachnologist Eugène Louis Simon, in recognition of his inception of Theridiosomatidae.

#### Diagnosis.

**Figure 1.**
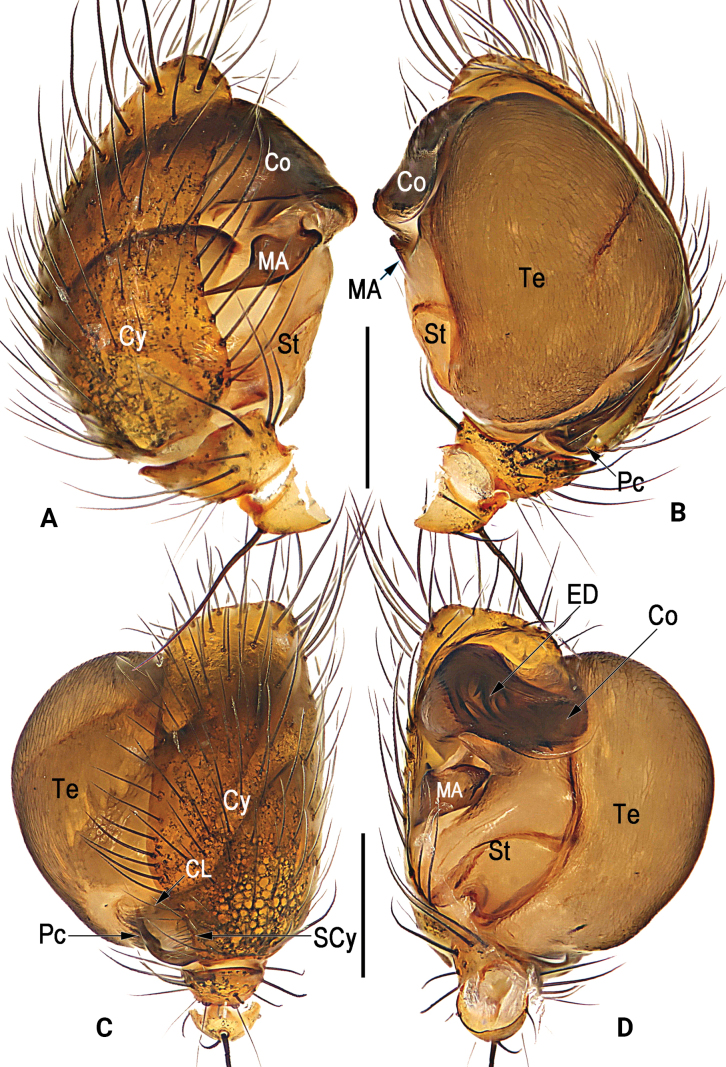
Male palp of *Simoniayouyiensis***A** prolateral **B** retrolateral **C** dorsal **D** ventral. Abbreviations: Co = conductor; CL = cymbial lobe; Cy = cymbium; ED = embolic division; MA = median apophysis; Pc = paracymbium; SCy = cymbial setae; St = subtegulum; Te = tegulum. Scale bars: 0.2 mm (**A–D**).

**Figure 2.**
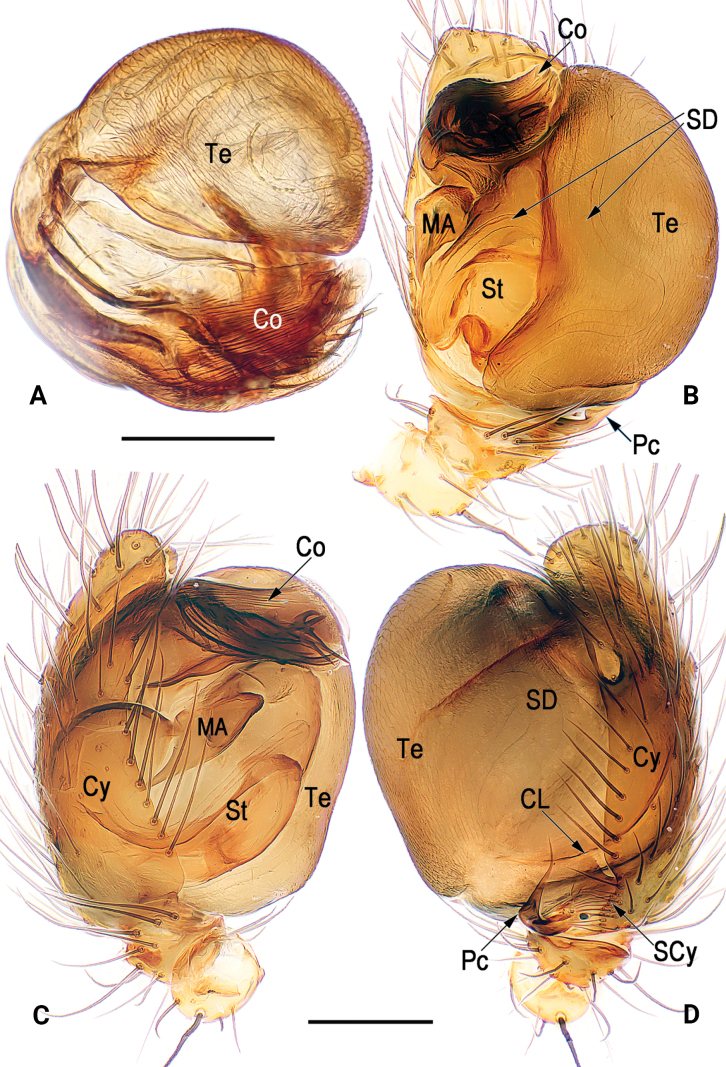
Bulb (**A**) and male palp (**B–D**) of *Simoniayouyiensis*, cited from Zhang and Lin (2016)**A** apical **B** ventral **C** prolateral **D** retrolateral. Abbreviations: Co = conductor; CL = cymbial lobe; Cy = cymbium; MA = median apophysis; Pc = paracymbium; SCy = cymbial setae; SD = sperm duct; St = subtegulum; Te = tegulum. Scale bars: 0.2 mm (**A–D**).

**Figure 3.**
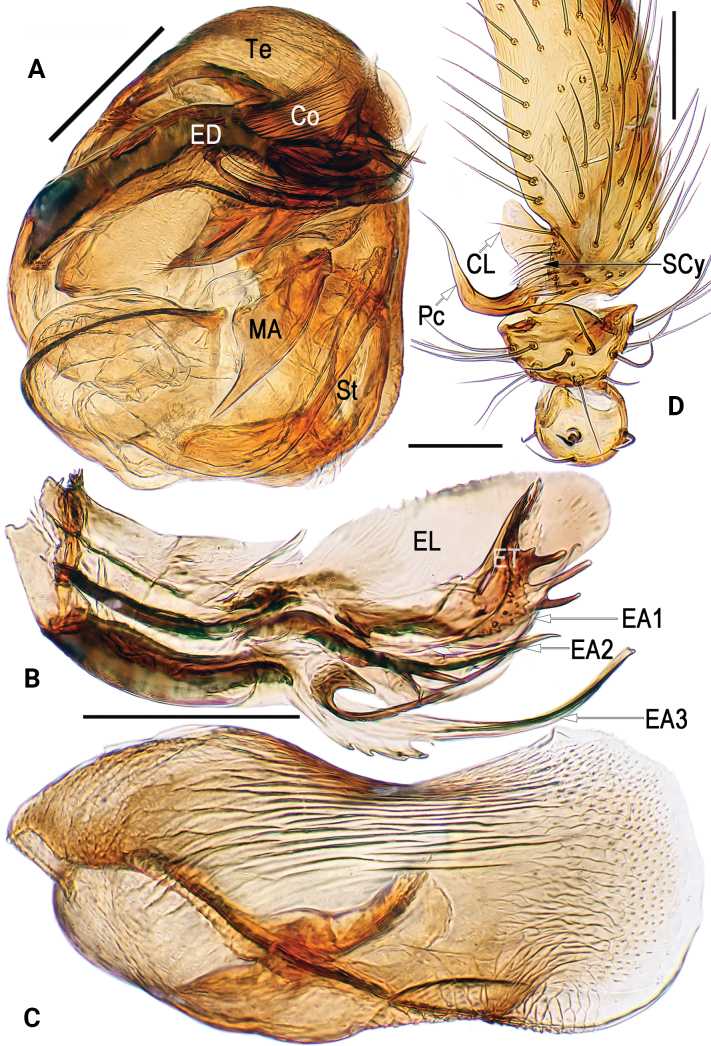
Male palpal sclerites of *Simoniayouyiensis*, cited from Zhang and Lin (2016)**A** bulb, prolateral **B** embolic division, ventral **C** conductor, ventral **D** cymbium, retrolateral. Abbreviations: Co = conductor; CL = cymbial lobe; EA = embolic apophysis; ED = embolic division; EL = embolic lobe; ET = embolic terminal; MA= median apophysis; Pc = paracymbium; SCy = cymbial setae; St = subtegulum; Te = tegulum. Scale bars: 0.1 mm (**A–D**).

**Figure 4.**
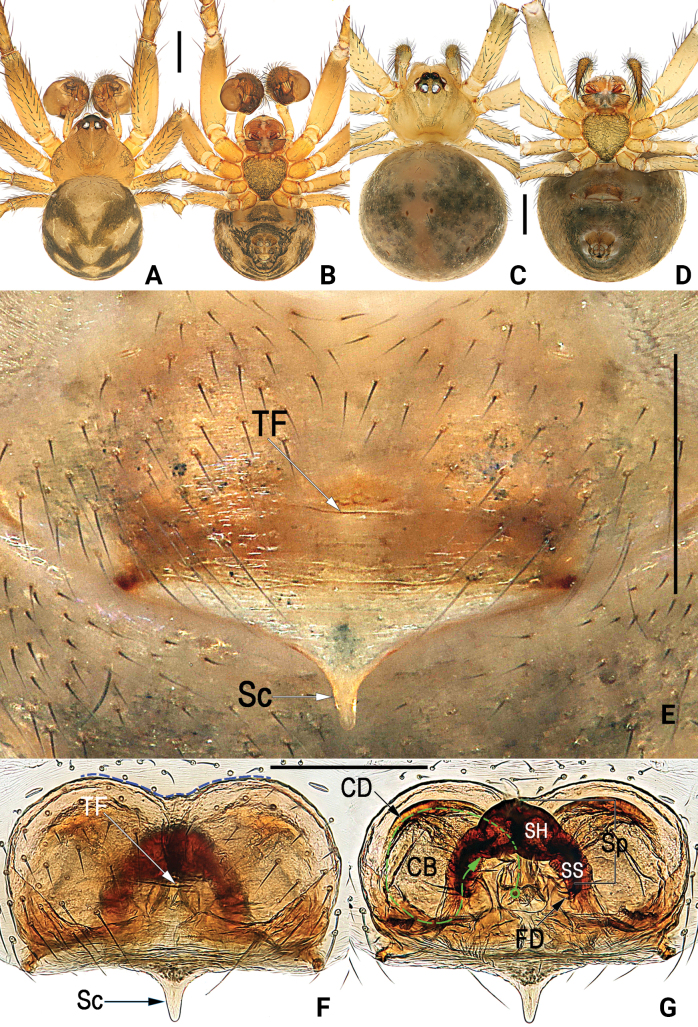
*Simoniayouyiensis*, male habitus (**A, B**), female habitus (**C, D**) and epigyne (**E–G**) **A** dorsal **B** ventral **C** dorsal **D** ventral **E** intact, ventral **F** cleared, ventral (blue dashed line showing the anterior margin of the epigynal plate) **G** cleared, dorsal (green dashed line showing schematic course of copulatory duct). Abbreviations: CB = copulatory bursa; CD = copulatory duct; FD = fertilization duct; Sc = scape; SH = spermathecal head; SS = spermathecal stalk; Sp = spermatheca; TF = transverse fold of epigynal plate. Scale bars: 0.5 mm (**A–D**); 0.2 mm (**E–G**).

**Figure 5.**
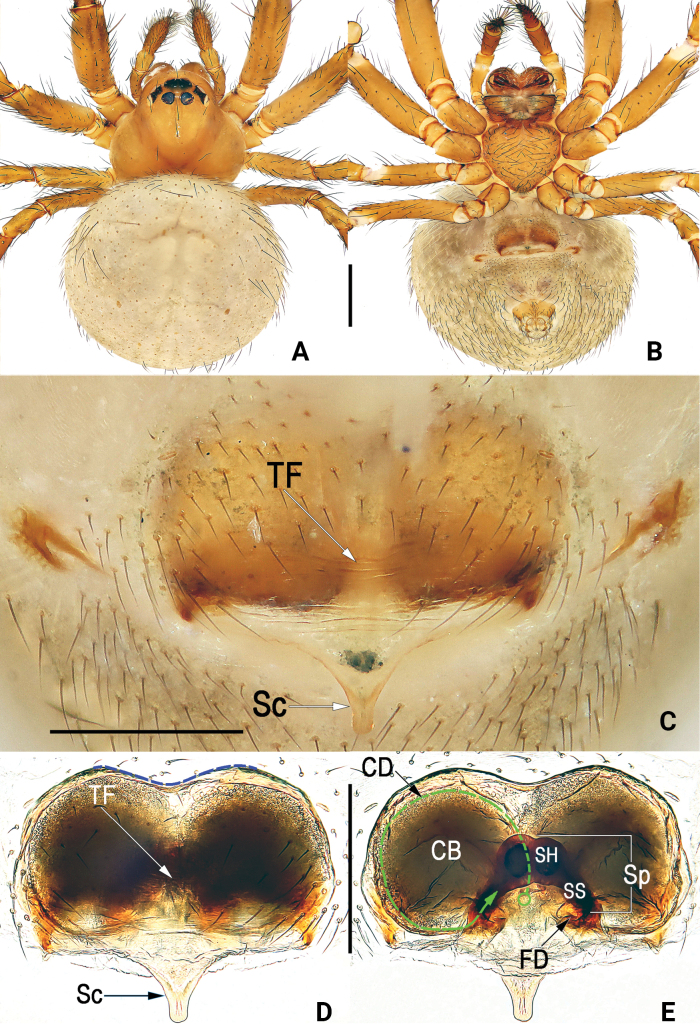
Holotype female of *Simoniasteineri* sp. nov., habitus (**A, B**) and epigyne (**C–E**) **A** dorsal **B** ventral **C** intact, ventral **D** cleared, ventral (blue dashed line showing the anterior margin of the epigynal plate) **E** cleared, dorsal (green dashed line showing schematic course of copulatory duct). Abbreviations: CB = copulatory bursa; CD = copulatory duct; FD = fertilization duct; Sc = scape; SH = spermathecal head; SS = spermathecal stalk; Sp = spermatheca; TF = transverse fold of epigynal plate. Scale bars: 0.5 mm (**A, B**); 0.2 mm (**C–E**).

**Figure 6.**
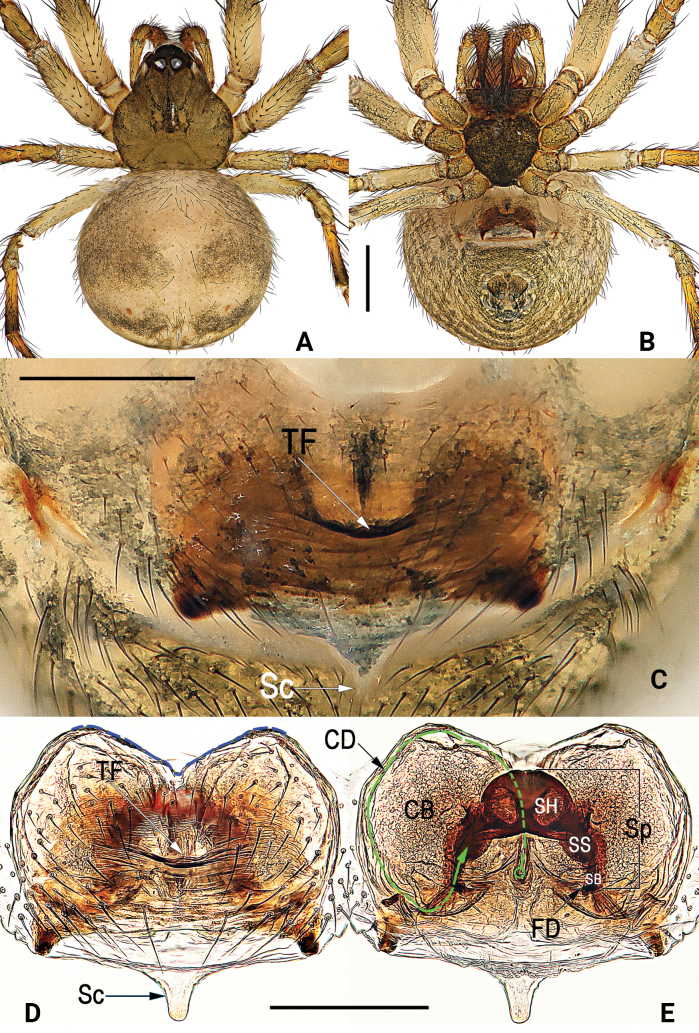
Holotype female of *Simoniasumatra* sp. nov., habitus (**A, B**) and epigyne (**C–E**) **A** dorsal **B** ventral **C** intact, ventral **D** cleared, ventral (blue dashed line showing the anterior margin of the epigynal plate) **E** cleared, dorsal (green dashed line showing schematic course of copulatory duct). Abbreviations: CB = copulatory bursa; CD = copulatory duct; FD = fertilization duct; SB = spermathecal base; Sc = scape; SH = spermathecal head; SS = spermathecal stalk; Sp = spermatheca; TF = transverse fold of epigynal plate. Scale bars: 0.5 mm (**A, B**); 0.2 mm (**C–E**).

*Simonia* gen. nov. resembles *Sennin* Suzuki, Hiramatsu & Tatsuta, 2022 in general shape of copulatory organs. Male palps of these two genera have similar embolic divisions with at least three bristle-like, sharp embolic apophyses; epigynes of both genera have similar spoon-shaped or equicrural triangular scape. *Simonia* gen. nov. can be distinguished from *Sennin* by a combination of following characters: cymbial outgrowth (cymbial apophysis) absent (vs present), conductor axe-shaped, almost hyaline and with vein-shaped grains (vs not axe-shaped, membranous, without vein-shaped grains), embolus long, extending to the distal part of embolic division, terminally torch-shaped, with a cylindric stalk and a multiramose apex (vs shorter and apex blunt, located at the proximal part of embolic division, embolic terminal absent), all embolic apophyses are not coiled (vs strongly curved or coiled), embolic division dorsally with large, hyaline lobe (vs embolic lobe absent) (cf. Figs 1–3 and Suzuki et al. 2022: figs 7, 9); epigynal plate surface distinctly wrinkled, with a distinctive transverse fold at midlength between anterior and posterior margins (vs surface slightly wrinkled, medially without the distinctive transverse fold), the anterior part of spermathecae fused (vs not fused, just only overlapped), copulatory duct indistinct, the course of the copulatory duct simple, forming a loop in the inside of copulatory bursa (vs distinct, and course more complex, with a coiled trajectory at the basal side of the spermathecae), copulatory bursa large, nearly as long as epigyne length (vs smaller or indistinct, barely longer than 1/2 length of epigynal plate) (cf. Figs 4E–G, 5C–E, 6C–E and Suzuki et al. 2022: figs 8, 10).

#### Description.

Small sized with body length 1.50–1.65 in males and 1.90–2.45 in females; carapace 0.83–0.96 long in males and 0.91–1.08 in females. Carapace nearly pyriform, in profile highest just behind ocular area, gradually sloping to pedicel, c. 1.35–1.45 times longer than high; carapace smooth, with long, sparse setae, yellow brown to dark brown, slightly darker anteriorly; cervical groove V-shaped, radial grooves and fovea indistinguishable. Sternum yellowish brown to dark, distinctly darker than carapace, clothed with dense setae, heart-shaped, anterior edge truncate, anterior and lateral margins with brown extensions fitting intercoxal concavities; posterior region strongly protruding between coxae IV. Female palp distally with erect, thin, dark bristles. Chelicerae slightly darker than carapace. Labium triangle shaped. Maxillae nearly trapezoidal, anterior edge straight, anterior and lateral margins slightly curved, slightly convergent posteriorly, with dense scopulae on inner margins. Legs long, uniformly coloured, slightly lighter than carapace, with darker femora and coxae I. Leg formula 1243. Abdomen ovoid, nearly as long as wide, abdominal colours and patterns variable; marginally clothed with sparse long setae, ventrally covered by fine short setae. Spinnerets brown. Male palp: same as in type species. Epigyne with small, hyaline scape (SC); epigynal plate surface distinctly wrinkled, with distinctive transverse fold (TF) at midlength between anterior and posterior margins; scape (SC) extending from posterior margin of epigynal plate, translucent; copulatory openings indistinct; copulatory duct (CD) indistinct, with simple course, forming loop in inside of copulatory bursa; spermathecae (Sp) consist of relatively large head (SS, anterior part) and slightly narrower stalk (SS, posterior part), and in addition with distinctly small base (SB, distal part) in *S.sumatra* sp. nov.; spermathecal heads fused, located centrally and juxtaposed; fertilization ducts (FD) acicular, membranous, located on basal surface of spermathecae; copulatory bursae (CB) represented spherical or oval sacs, large, nearly as long as epigyne length, surface hyaline, wrinkled and ribbed, bursae touching each other.

#### Composition and distribution.

*Simoniayouyiensis* (Zhao & Li, 2012) from Laos, Vietnam and Guangxi in China, *S.steineri* sp. nov. from Laos, and *S.sumatra* sp. nov. from Sumatra.

#### Comments.

A preliminary genus-level taxonomic molecular analysis of Theridiosomatidae from Southeast Asia was carried, based on five targeted genes (two mitochondrial genes: 16S and COI; three nuclear genes: 18S, 28S, and H3). According to the results (unpublished): (1) the monophyly of the genus *Simonia* gen. nov. is supported; (2) this new genus is related to two genera exclusively distributed in SE Asia, *Karstia* and *Sennin*. Morphologically, the new genus is also similar to *Karstia*, but can be distinguished by the absence of cymbial outgrowth, presence of torch-shaped embolic terminal and large, hyaline embolic lobe, and by the fused anterior parts of the spermathecae, large copulatory bursa, as well as a set of other characters of the copulatory organs (see diagnosis of the genus above and key to theridiosomatid genera endemic to Oriental Realm below).

The type species of *Simonia* gen. nov., *S.youyiensis* was originally assigned to the *Baalzebub*, although it did not show typical *Baalzebub* features. *Baalzebub* is definitely not monophyletic. There is a strong possibility that *Baalzebubsensu stricto* contains only two species from the Neotropical Realm: *B.baubo* and *B.albonotatus* (Petrunkevitch, 1930). These Neotropical *Baalzebub* species share the following distinctive suite of characters, here contrasted with the corresponding condition in *Simonia* gen. nov.: 1) embolic apophyses spatulate, thick and blunt (vs bristle-like and slender, apically sharp); 2) embolus claw-shaped, not branched (vs torch-shaped, with a cylindric stalk and a multiramose apex); 3) embolic lobe absent (vs present); 4) conductor not axe-shaped, surface smooth (vs axe-shaped, surface with many vein-shaped grains); 5) scape large, at least longer than 1/2 length of epigynal plate (vs small, no more than 1/4 length of epigynal plate); 6) epigynal plate surface smooth (distinctly wrinkled, with a distinctive transverse fold); and 7) copulatory bursa surface smooth, small, less than 1/2 length of epigynal plate (surface wrinkled and ribbed, large, as long as length of epigyne) (cf. Coddington 1986: figs 161–164, 183, 184, 186, 187 and Figs 1–3, 4E–G, 5C–E, 6C–E). In view of the above-mentioned facts, it is currently impossible to discern any obvious derived features that could indicate a close relationship between *S.youyiensis* and the genus *Baalzebubsensu stricto*, leaving no doubts that our new combination and the establishment of a new genus are correct.

### ﻿Key to theridiosomatid genera endemic to Oriental Realm (males)

**Table:** 

1	Embolic division complex and broad, with multiple embolic apophyses (Figs 1A, B, D, 2A, B, C, 3A, B)	**2**
–	Embolic division simple and thin, not branched or slightly forked distally	**4**
2	Embolic apophyses blunt and spatulate	***Karstia* Chen, 2010**
–	Embolic division with at least three bristle-like and sharp embolic apophyses (Fig. 3B)	**3**
3	Cymbial outgrowth absent; embolus terminally shaped like torch, with cylindric stalk and multiramose apex (Fig. 3B); embolic apophyses not coiled (Fig. 3B); embolic division dorsally with large, hyaline lobe (Fig. 3B)	***Simonia* Yu & Lin, gen. nov.**
–	Cymbial outgrowth present; embolus terminally blunt; all embolic apophyses strongly coiled; embolic lobe absent	***Sennin* Suzuki, Hiramatsu & Tatsuta, 2022**
4	Embolus claw-shaped, extremely simple, shorter than 1/2 width of tegulum	***Menglunia* Zhao & Li, 2012**
–	Embolus whip-shaped, longer than tegulum width	**5**
5	Cymbium elongate, with ventral groove; conductor disc-shaped; embolic apophysis distinctly long, filiform; median apophysis no more than 1/4 length of tegulum	**6**
–	Cymbium unmodified; conductor tubular; embolic apophysis absent; median apophysis at least 1/3 length of tegulum	**7**
6	Median apophysis square shaped	***Tagalogonia* Labarque & Griswold, 2014**
–	Median apophysis ovoid, elongated and distally acute	***Coddingtonia* Miller, Griswold & Yin, 2009**
7	Palpal tibia with retrolateral apophysis; embolic distal end not forked	***Sinoalaria* Zhao & Li, 2014**
–	Retrolateral tibial apophysis absent; embolic distal end forked	***Chthonopes* Wunderlich, 2011**

### ﻿Key to theridiosomatid genera endemic to Oriental Realm (females)

**Table:** 

1	Spermathecae completely separated	**2**
–	Spermathecae touching each other, overlapping or partially fused (Figs 4G, 5E, 6E)	**5**
2	Epigynal plate centrally with deep transversal pit; copulatory duct forming at least three loops around spermathecae	**3**
–	Epigynal plate centrally without pit; copulatory duct not as above	**4**
3	Central pit located anteriorly to spermathecae, which are visible through integument, separated from anterior margin of epigynal plate by not more than 1/2 length of epigynal plate; lateral wings of copulatory bursae hyaline, slightly sclerotized, round, swollen with dorso-median gland ductules	***Coddingtonia* Miller, Griswold & Yin, 2009**
–	Central pit located posteriorly to spermathecae, separated from anterior margin of epigynal plate by c. 2/3 length of epigynal plate; lateral wings membranous and soft, not swollen, without gland ductules	***Tagalogonia* Labarque & Griswold, 2014**
4	Epigyne with scape; vulval center with V-shaped medial structure and with accessory spermathecae	***Chthonopes* Wunderlich, 2011**
–	Scape, V-shaped medial structure and accessory spermathecae absent	***Menglunia* Zhao & Li, 2012**
5	Vulva centrally with U-shaped medial structure; copulatory ducts rise and curl up to form two folds	***Sinoalaria* Zhao & Li, 2014**
–	Medial structure lacking; conformation of copulatory ducts not as above	**6**
6	Epigynal plate surface smooth; scape shaped like equilateral triangle, longer than 1/2 length of epigynal plate, with two straight lateral margins and sharp apex	***Karstia* Chen, 2010**
–	Epigynal plate surface wrinkled; scape spoon-like, or shaped like acute triangle, no more than 1/3 length of epigynal plate, apex blunt, lateral margins slightly curved (Figs 4E, F, 5C, D, 6C, D)	**7**
7	Anterior part of spermathecae fused (Figs 4G, 5E, 6E); copulatory duct indistinct, course of copulatory duct simple, forming loop inside of bursa (Figs 4G, 5E, 6E); bursa distinctly large, nearly as long as epigyne length (Figs 4G, 5E, 6E)	***Simonia* Yu & Lin, gen. nov.**
–	Anterior part of spermathecae not fused, just only touching or overlapping; copulatory duct distinct, course of copulatory duct complex, with coiled trajectory at basal side of spermathecae; bursa smaller, barely longer than 1/2 length of epigynal plate	***Sennin* Suzuki, Hiramatsu & Tatsuta, 2022**

### ﻿Key to species of *Simonia* gen. nov. (females)

**Table:** 

1	Dorsum of abdomen uniformly colored (Fig. 5A)	***S.steineri* sp. nov.**
–	Abdomen with dorsal pattern (Figs 4C, 6A)	**2**
2	Bursae spherical (Fig. 4G)	***S.youyiensis***
–	Bursae egg-shaped (Fig. 6E)	***S.sumatra* sp. nov.**

### ﻿ Simonia youyiensis

Taxon classificationAnimaliaAraneaeTheridiosomatidae

(Zhao & Li, 2012) comb. nov.

00C66505-BCF8-5CFB-825C-C345C05BB383

**Figure 7.**
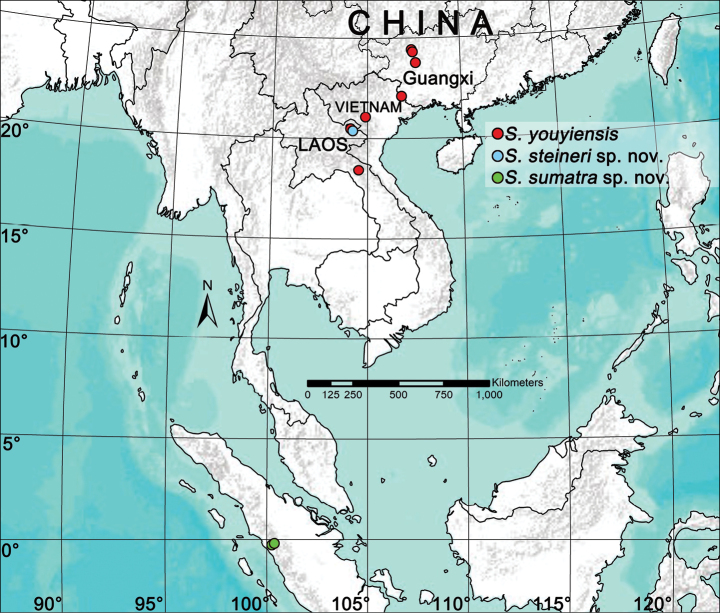
Distribution records of *Simonia* gen. nov. species.

Figs 1 , 2 , 3 , 4 , 7

Baalzebub youyiensis Zhao & Li, 2012: 17, figs 9A–E, 10A, B (♀); Zhang and Lin 2016: 222, figs 1–17 (♂♀).

#### Type material.

***Holotype*** ♀ and ***paratypes*** 3♀ (IZCAS), **China**: Guangxi, Pingxiang City, Youyi Town, Bantou Vill., Niuyan Cave, 22°05.666'N, 106°45.439'E, 251 m, 18.I.2011, Z. Chen and Z. Zha leg. Examined.

#### Other material examined.

**China**: 1♂ 12♀ (IZCAS), Guangxi Prov., Baise City, Pingguo Co., Liming Vill., Ganmoyan Cave, 23°48.330'N, 107°31.526'E, 22.IX.2015, J. Wu and Z. Chen leg.; 1♂ 7♀ (NHMSU), Guangxi Prov., Hechi City, Donglan Co., Sanshi Town, Gongping Vill., a nameless cave, 24°21.347'N, 107°23.190'E, 368 m, 9.II.2015, Y. Li and Z. Chen leg. **Laos**: 3♂ 17♀ (NHMSU), Bolikhamsai Prov., Lak Sao City, Transiten, Lang Cave, 18°23.318'N, 104°32.675'E, 318 m, VIII.2012, P. Jäger leg. **Vietnam**: 1♂ 3♀ (IZCAS), Phú Tho Prov., Thanh Son Dist., Xuan Son Commune, Xuan Son National Park, Lang Cave, 21°06.600'N, 104°57.600'E, 375 m, 26.X.2012, H. Zhao and Z. Chen leg.

#### Diagnosis.

Females of *S.youyiensis* are most similar to those of *S.sumatra* sp. nov. by having similar habitus and general shape of the vulva. *Simoniayouyiensis* differs from *S.sumatra* sp. nov. in 1) anterior margin of the epigynal plate slightly concaved (c. 140°) (vs concaved c. 120°) (cf. Fig. 4E, F and Fig. 6C, D), 2) spermathecae comma-shaped (vs spermathecae shaped like the whole hind leg of a frog) (cf. Fig. 4G and Fig. 6E), and 3) bursae spherical (vs egg-shaped) (cf. Fig. 4G and Fig. 6E).

#### Description.

**Male** (Fig. 4A, B): Carapace brown, slightly darker in ocular region, without distinct pattern; cervical groove and radial grooves indistinct. AER distinctly recurved, PER slightly procurved. Sternum yellowish brown. Mouthparts reddish brown. Legs uniformly yellowish. Abdomen round, dorsum basically light brown, with 3 pairs of black diagonal bands on sides, forming 3 V-shaped stripes; venter centrally with black trapezoidal speckle, marginally with several discontinuous streaks. ***Measurements***: Total length 1.62. Carapace 0.83 long, 0.81 wide. Clypeus 0.21 high. Sternum 0.52 long, 0.49 wide. Abdomen 1.14 long, 1.08 wide. Length of legs: I 3.32 (1.01, 0.39, 0.88, 0.63, 0.41); II 2.51 (0.81, 0.32, 0.58, 0.51, 0.29); III 1.84 (0.52, 0.21, 0.38, 0.41, 0.32); IV 2.22 (0.49, 0.32, 0.52, 0.58, 0.31).

Palp (Figs 1–3): Femur unmodified. Patella round and small, nearly as long as tibia, dorsally bears strong and long macroseta slightly longer than tibia. Tibia cup-shaped, about 1/6–1/5 length of cymbium, with several sparse setae. Cymbium (Cy) broad, navicular, c. 2.1 × longer than wide, dorsally clothed with dense setae, basolaterally with lobe (CL) and row of longitudinally arranged setae (SCy) along suture between cymbium and lobe. Cymbial lobe represented by small and semi-transparent sheet, just like outline of thumb. Paracymbium (Pc) L-shaped, about 1/5–1/4 length of cymbium, with sharp and spine-like tip. Tegulum (Te) capacious and inflated, c. 1.5 × longer than wide, surface with countless delicate texture; sperm duct (SD) indistinct and sinuate. Subtegulum (ST) located prolaterally to tegulum, represented by large and surface smooth tubercle, slightly longer than 1/2 length of tegulum. Median apophysis (MA) small, about 2/5 length of tegulum, shaped like tadpole, consisting of relatively wide head (distal part) and narrow tail (proximal part); distal process blunt, apex round and rough, with many tiny and scale-like tooth; proximal process triangular, gradually narrowing toward its apex, apex sharp. Conductor (Co) large and axe-shaped, aligned transversely on anterior part of bulb; almost all of conductor hyaline and with vein-shaped grains, except membranous distal margin. Embolic division nearly as long as conductor, hidden behind conductor, consisting of broad embolic lobe (EL), embolic terminal (ET), and at least 3 apophyses (EA); embolic terminal short, less than 1/2 length of embolic lobe, shaped like a torch, with cylindric stalk and multiramose apex; all embolic apophyses bristle-like and slender, slightly longer than embolic terminal; some apexes of embolic terminal and embolic apophyses overpass distal margin of conductor.

**Female.** Somatic features as in Fig. 4C, D and coloration distinctly lighter than in male. ***Measurements***: Total length 2.40. Carapace 0.91 long, 0.90 wide. Clypeus 0.20 high. Sternum 0.53 long, 0.51 wide. Abdomen 1.56 long, 1.27 wide. Length of legs: I 3.81 (1.31, 0.42, 0.78, 0.89, 0.41); II 3.17 (0.87, 0.43, 0.81, 0.68, 0.38); III 2.03 (0.63, 0.28, 0.37, 0.44, 0.31); IV 2.32 (0.62, 0.31, 0.49, 0.57, 0.33).

Epigyne (Fig. 4E–G). Epigynal plate large, c. 1.4 × wider than long, anteriorly slightly concaved by c. 140°, arrangement of various parts of vulva indistinctly visible through tegument. Scape (Sc) T-shaped, small, about 1/5 length of epigynal plate. Spermathecae (Sp) comma-shaped, with bean-shaped head (SH) and slightly curved stalk (SS); anterior surface of spermathecal heads touching anterior margin of epigynal plate; spermathecal stalks extending obliquely, separated by about 2–3× diameters of stalks. Copulatory bursae (CB) spherical.

#### Distribution.

China (Guangxi), Laos, Vietnam (Fig. 7).

### ﻿ Simonia steineri

Taxon classificationAnimaliaAraneaeTheridiosomatidae

Yu & Lin sp. nov.

A7BED778-6490-5381-8E04-54CF1C6F4865

https://zoobank.org/98BD9ACB-E3FC-42A8-B6E1-FD778E841EFE

Figs 5 , 7

#### Type material.

***Holotype*** ♀ (IZCAS) and ***paratype*** 1♀ (IZCAS), **Laos**: Hua phan Pro., Guesthouse cave, 20°24.176'N, 104°13.818'E 13.I.2008, H. Steiner leg.; ***paratype*** 1♀ (NHMSU), Hua phan Pro., Tham Kuong Tai, 20°27.470'N, 104°09.850'E, 12.I.2014, H. Steiner leg.

#### Etymology.

The specific name is a patronym after Mr. Helmut Steiner (Hessen, Germany), collector of the type series.

#### Diagnosis.

The females of *S.steineri* sp. nov. can be easily distinguished from other congeners by the following characters: 1) dorsum of abdomen uniformly coloured (vs with pattern) (cf. Fig. 5A and Figs 4C, 6A); 2) spermathecae separated from anterior margin of epigynal plate by more than 1.5 × diameters of spermathecal head (vs nearly touching anterior margin) (cf. Fig. 5E and Figs 4G, 6E); and 3) spermathecae hammer-shaped, spermathecal heads globular, spermathecal stalks straight (vs spermathecae not hammer-shaped, spermathecal heads bean-shaped, spermathecal stalks slightly curved) (cf. Fig. 5E and Figs 4G, 6E).

#### Description.

**Female** (Fig. 5A, B): Carapace, uniformly brown except a V-shaped dark brownish band along cervical groove; cervical groove and radial grooves indistinct. AER distinctly recurved, PER almost straight in dorsal view. Sternum deep brown. Mouthparts proximally reddish brown, distally light brown, inner margins white. Legs uniformly yellowish. Abdomen round, marginally clothed with sparse long setae, uniformly yellowish white, without any markings or distinct pattern. ***Measurements***: Total length 2.12. Carapace 1.03 long, 0.86 wide. Clypeus 0.21 high. Sternum 0.55 long, 0.52 wide. Abdomen 1.46 long, 1.37 wide. Length of legs: I 3.12 (0.98, 0.32, 0.93, 0.58, 0.31); II 3.03 (0.92, 0.28, 0.81, 0.69, 0.33); III 2.42 (0.73, 0.27, 0.58, 0.63, 0.21); IV 2.93 (0.89, 0.32, 0.77, 0.64, 0.31).

Epigyne (Fig. 5E–G). Epigynal plate c. 1.2 × wider than long, anteriorly concaved by c. 140°, copulatory bursae distinctly visible through integument. Scape (Sc) shaped like an acute triangle, relatively large, about 1/4 length of epigynal plate. Spermathecae (Sp) hammer-shaped, with globular-shaped heads (SH) and columnar stalk (SS); anterior surface of spermathecae separated from anterior margin of epigynal plate by more than 1.5 × diameters of spermathecal head; spermathecal stalks straight, extending obliquely, separated by about 3–4× diameters of stalks. Bursae (CB) subglobular.

#### Distribution.

Known only from the type locality, Guesthouse cave, Huapan Province, Laos (Fig. 7).

### ﻿ Simonia sumatra

Taxon classificationAnimaliaAraneaeTheridiosomatidae

Yu & Lin sp. nov.

48EC40E7-92B9-55EE-BAB9-F42461E334FA

https://zoobank.org/25562397-6C9F-49E3-BF7D-0C50DF7334CA

Figs 6 , 7

#### Type material.

***Holotype*** ♀ and ***paratype*** 1♀ (IZCAS), **Indonesia**: West Sumatra Prov., Kab Agam Co., Kecamatan Matur district, 0°15.739'S, 100°18.491'E, 01.XII.2013, H. Zhao leg. ***Paratypes*** 1♀ and 1juv. (NHMSU), West Sumatra Prov., Kab Agam Co., Simarasok Vill., Cua Luwuang, 0°14.902'S, 100°28.993'E, 710 m, 11.I.2014, H. Zhao leg.

#### Etymology.

The species name is derived from the type locality; noun in apposition.

#### Diagnosis.

The female of the new species resembles those of *S.youyiensis* in having similar habitus and general appearance of the epigyne (cf. Fig. 6 and Fig. 4C–G), but can be easily distinguished by 1) anterior margin of epigynal plate concaved c. 120° (vs c. 140°) (cf. Fig. 6C, D and Fig. 4E, F), 2) spermathecae shaped like the whole hind leg of a frog, consisting of head, stalk and base (vs comma-shaped, only with head and stalk) (cf. Fig. 6E and Fig. 4G), and 3) bursae egg-shaped (vs spherical) (cf. Fig. 6E and Fig. 4G).

#### Description.

**Female** (Fig. 6A, B): Carapace brownish posteriorly, distinctly darker anteriorly and marginally, with distinct pattern on pars cephalica consisting of pair of dark lateral bands and Ψ-shaped markings behind posterior eyes, markings starting from behind PME and PLE almost reaching dark fovea. AER distinctly recurved, PER distinctly recurved in dorsal view. Sternum uniformly black. Mouthparts coloured as sternum. Legs uniformly yellowish white. Abdomen round, covered with sparse long setae; dorsum basically black, centrally with 2 pairs of muscular depressions, anteriorly with pair of large, nearly fan-shaped patches, posteriorly with pair of √-shaped band; venter slightly darker than dorsum, without distinct pattern. ***Measurements***: Total length 2.28. Carapace 0.96 long, 0.88 wide. Clypeus 0.22 high. Sternum 0.48 long, 0.50 wide. Abdomen 1.56 long, 1.28 wide. Length of legs: I 2.71 (1.02, 0.33, 0.57, 0.51, 0.28); II 2.42 (0.83, 0.27, 0.52, 0.49, 0.31); III 1.74 (0.48, 0.21, 0.41, 0.41, 0.23); IV 2.29 (0.91, 0.27, 0.42, 0.41, 0.28).

Epigyne (Fig. 6E–G). Epigynal plate c. 1.2 × wider than long, anteriorly concaved by c. 120°. Scape (Sc) spoon-shaped, small, about 1/6 length of epigynal plate. Spermathecae (Sp) shaped like the whole hind leg of a frog, with bean-shaped head (SH), conical stalk (SS) and distinctly narrowed base (SB); anterior surface of spermathecae touching anterior margin of epigynal plate; spermathecal stalks and bases extending obliquely, widely separated by c. 1/2 width of epigynal plate. Bursae (CB) egg-shaped.

#### Distribution.

Known only from the type locality, West Sumatra, Indonesia (Fig. 7).

## Supplementary Material

XML Treatment for Simonia

XML Treatment for Simonia youyiensis

XML Treatment for Simonia steineri

XML Treatment for Simonia sumatra

## ﻿Additional information

### Author ORCIDs

Jianshuang Zhang https://orcid.org/0000-0003-4010-3082

Hao Yu https://orcid.org/0000-0002-9113-2425

Yucheng Lin https://orcid.org/0000-0002-5054-0633

### Data availability

All of the data that support the findings of this study are available in the main text.
